# Preventing root caries development under oral biofilm challenge in an artificial mouth

**DOI:** 10.4317/medoral.18768

**Published:** 2013-03-25

**Authors:** May L. Mei, Chun H. Chu, Edward CM. Lo, Lakshman P. Samaranayake

**Affiliations:** 1 BDS, MDS, PhD Faculty of Dentistry, The University of Hong Kong

## Abstract

Objectives: To study the preventive effects of chlorhexidine against root caries under oral biofilm in an artificial mouth. Study Design: Sixteen human tooth-root disks were inoculated with a salivary sample that was produced by mixing the unstimulated saliva of three adults who had no untreated caries. The disks were incubated in an artificial mouth fed with a 5% sucrose solution three times daily for one week. Eight disks received a twice daily rinse of 0.12% chlorhexidine (test group). The other eight disks were rinsed in distilled water (control). The biofilm was then studied with three techniques: colony forming unit (CFU) counting, scanning electron microscopy (SEM) and confocal laser scanning microscopy (CLSM). The changes in the chemical structure of the root surface were studied using Fourier transform infra-Red spectroscopy. Type-I collagen and proteoglycans on the root surface were quantified using immunocytochemical staining. Results: The log CFU for the test and control groups were 4.21 and 8.27, respectively (p<0.001). The CFU count of Streptococci and Lactobacilli were negligible. Both the SEM and the CLSM showed suppressed bacteria growth in the test group. The log [amide-I: HPO42-] of the test and control groups were 1.11 and 1.93, respectively (p=0.02). The mean counts of sound type-I collagen in the test and control groups were 16.8/?m2 and 13.0/?m2, respectively (p<0.001), whereas the mean counts of intact proteoglycans were 5.6/?m2 and 3.5/?m2, respectively (P<0.001). Conclusions: Chlorhexidine suppressed the growth of selected cariogenic bacteria in oral biofilm on the root surface and thus protected tooth-root from cariogenic challenge.

** Key words:**Chlorhexidine, biofilm, caries risk, root, caries, artificial mouth, demineralization, streptococci, lactobacilli, proteoglycans, collagen I.

## Introduction

The proportion of older people in the population is growing and their inadequate plaque control procedures make root caries an increasingly common dental health problem (1). Soft tissue recession due to age, traumatic toothbrushing habits, periodontal disease or periodontal treatment will unavoidably result in more tooth root surfaces that are at risk for the development of root caries (2). Restorative treatment of root caries is notoriously difficult. Post-treatment pain and hypersensitivity are very common, and this may contribute to increased tooth loss in many people with root caries. Therefore, prevention of root caries is an important issue in clinical practice.

Fluoride agents have been used in dental caries management (3). In addition, Clinical experience and population-based studies have demonstrated that mechanical plaque control methods can maintain good oral hygiene and oral health. However, these plaque control methods require good manual dexterity and can be difficult to implement in certain circumstances, such as tooth hypersensitivity or among older patients (4). In these circumstances, antimicrobial agents may serve as a valuable complement to mechanical plaque removal (5). Chlorhexidine is one of the commonly used antimicrobial agents in the management of caries and periodontal diseases. Its popularity is not only due to its broad antimicrobial spectrum, which includes Gram-positive and Gram-negative bacteria, but also due to its retention in the mouth, which prolongs its antimicrobial effect. The uptake of chlor-hexidine by bacteria has been shown to be extremely rapid, with a maximum effect occurring in a very short time of around 20 seconds (6). Nevertheless, Autio-Gold (7) reviewed the chlorhexidine literature and found that its action on dental plaque or oral biofilm is inconclusive. Additional factors such as the strain of bacteria in the oral biofilms (8), the growth condition of the oral biofilm (9) and the nature of the bacteria in the substratum (10) might affect the efficacy of chlorhexidine. Further studies are thus necessary to study the anti-caries effect of chlorhexidine.

A recent systemic review concluded that the development of caries on the root surface is associated with the composition and quantity of dental plaque, diet, the composition and flow of saliva, and exposure to fluoride (2). Laboratory studies were conducted to anti-caries effect of chlorhexidine on single species biofilms (11,12) in simplified environment. It is noteworthy that bacteria in biofilms are notably less susceptible to chlorhexidine than when grown in batch culture. Many bacteria living together in a biofilm experience stress. This implies that their stress defense mechanisms are turned on and thus they are even less susceptible to chlorhexidine. Dental plaque is a complex multi-species biofilm where bacteria communicate, protect one another and form a strong resistance to anti-microbial. Study using a complex multi-species biofilm generated from oral cavity should be more realistic approach to evaluate anti-microbial effect of chlorhexidine (13). Furthermore, it is also more desirable to study oral biofilms in an artificial mouth that closely simulates oral environment such as salivary flow, redox potential, acidity and temperature. This experiment was therefore carried out to study the effects of chlorhexidine on tooth root surface challenged with oral biofilm developed from human saliva in a sophisticated artificial mouth.

## Material and methods

-Tooth disk preparation

This study was approved by the Institutional Review Board (IRB-UW08-952) and patient consent was obtained before the study. Diamond trephine was used to prepare 16 tooth root disks with 5mm diameters from 16 extracted sound human molars. Half of tooth root disks’ surfaces were covered with nail vanish (Clarins, Paris, France) as an internal control. The remaining surfaces of the tooth root disks were covered with varnish. The tooth disks were sterilized with ethylene oxide for 16 hours (14).

-Oral bacteria sampling

The method of creating an oral bacteria sample was adapted from Navazesh (15). Three healthy middle aged patients (35 to 44 years old) attending a dental hospital were recruited for saliva collection. They had no clinically detectable caries, no periodontal disease with pocket depth 4mm or above, no salivary gland disease or dysfunction, no systemic diseases and they were not taking medication. They had abstained from their oral hygiene practice for 24 hours prior to saliva collection. Each patient was asked to spit out unstimulated saliva. Five ml of saliva was collected from each patient and then the saliva samples collected from the three patients were mixed together and centrifuged for 10 min at 3,000 rpm. After centrifugation, cell pellets were harvested and washed three times with 1% phosphate buffered saline (PBS). The washed pellets were re-suspended in PBS for bacteria adhesion.

-Formation of oral biofilms in the artificial mouth

**Figure 1:**
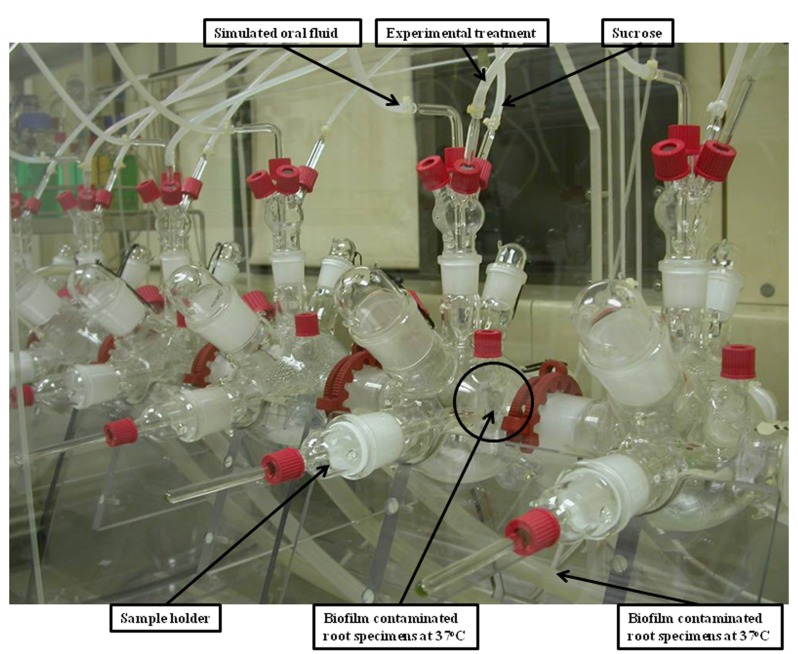
Configuration of the artificial mouth.

Each root disk was inoculated with a 300µL aliquot of bacteria and was then inserted into the artificial mouth for bacteria adhe-sion. The artificial mouth is shown in figure 1. A humidified gas mixture of 5% carbon dioxide and 95% nitrogen was supplied continuously at 60ml/min. The temperature inside the incubator was maintained at 37ºC. Simulated oral fluid defined medium mucin (DMM) was continuously supplied at 0.06 mL/min to simulate the salivary flow (16). A sucrose solution at 5% was sup-plied for six minutes with a flow rate of 15 mL/hr monitored by a computer program (LabVIEW® software Version 2.2). The sucrose supply was delivered every eight hours to simulate a real life dietary situation. The inoculated bacteria were allowed to grow and form a biofilm on the surface of the tooth disks in the artificial mouth for seven days.

-Group assignment

The 16 disks were randomly divided into test and control groups. Throughout the seven day test period, 0.12% chlorhexidine gluconate was supplied at a flow rate of 0.25 mL/min for six minutes every twelve hours to the test group of tooth root blocks in the artificial mouth. Distilled water was used in the control group.

-Biofilm study 

After the seven-day experiment, the oral biofilm was collected from the tooth root blocks. The Growth Kinetic of the generated biofilm was assessed by Colony Forming Unit (CFU) counting. The total CFU was counted in a blood agar plate and individual species were counted in selective medium plates. Selective media agar plates of Mitis Salicarius, Rogosa and Actinomyces were used for Streptococci, Lactobaciili and Actinomycetes spp., respectively. Topographical features of the biofilm were observed using scanning electron microscopy (SEM) (Leo 1530 Gemini, Oberkochen, Germany) at 12 kV in high-vacuum mode (17). The viability of the biofilm was studied using confocal laser scanning microscopy (CLSM) (Fluoview FV 1000, Olympus, Tokyo, Japan). The biofilms were labelled using two fluorescent probes, specifically propodium iodide (PI) and SYTO-9 (LIVE/DEAD BacLight Bacterial viability kit, Molecular Probes, Eugene, OR, United States). PI specifically labels the dead cells in red, whereas live cells are labelled green by STYO-9. Cellular images of the biofilms were performed using CLSM (Fluoview FV 1000, Olympus, Tokyo, Japan) (19). Four images of each biofilm specimen were obtained using CLSM (Fluoview FV 1000, Olympus, Tokyo, Japan) and examined using special image analysis software (Image J; National Institutes of Health, USA). The red-to-green (dead-to-live bacteria) ratio was calculated to indicate the anti-microbial effect of the therapeutic agent (18).

-Hard tissue study

Each tooth root disk was sectioned longitudinally to create thin sections that were each approximately 120 µm thick. The changes in the chemical structure of the root surface were studied using Fourier transform infra-red spectroscopy (FTIR) (UMA-500 machine, Bio-Rad Laboratories, CA, USA), and the amount of type I collagen and proteoglycans were quantified using immunocytochemical staining. In the FTIR analysis, the mineral content was calculated on the basis of the spectrally derived matrix-to-mineral ratio (the integrated area of protein amide I peak between 1585-1720 cm-1 and phosphate (HPO42-) peak between 900 and 1200 cm-1) from an area of 100 × 100 µm on the surface of the root blocks. The varnished part of root surface of the disk was used as an internal control.

After the FTIR assessment, the six thin sections were then ultrasonicated for one minute in de-ionized water (pH 7.4) and exposed to 10% citric acid for 15 seconds to decalcify the surfaces of the sections. A double-immunolabelling procedure was performed with two monoclonal primary antibodies: an IgG anti-type I collagen and an IgM anti-chondroitin 4/6 sulphate (mouse monoclonal; Sigma Chemical Co., St. Louis, MO, USA). They were used for the simultaneous detection of the distribution of antigenically intact collagen fibrils and proteoglycans. Gold labeling was performed with two secondary antibodies conjugated with gold particles of different sizes: an IgG goat anti-mouse-IgG conjugated with 30-nm gold particles (British BioCell International) for type I collagen identification, and an IgG goat anti-mouse-IgM conjugated with 15 nm colloidal gold for chondroitin 4/6 sulphate identification (British BioCell International). The specimens were prepared and examined under SEM (Leo 1530, Oberkochen, Germany) at 20 kV. Images with the same magnification were obtained for each section (n=6 in each group). The labeling index was calculated as the mean of the gold particle number/?m2 in the visible organic network obtained from each image (19).

-Statistical Analysis

Analyses were performed with SPSS 17.0 software (SPSS Inc., Chicago, IL, USA). A parametric t test was used to detect differ-ences between the treatment and control groups in log CFU value and log [amide I: HPO42-] value, and between the mean num-ber of 30 nm of colloidal labeling type I collagen and the 15 nm particles labeling proteoglycans. A 5% statistical significance level was used for all analyses.

## Results

-Oral biofilm characteristics

**Figure 2:**
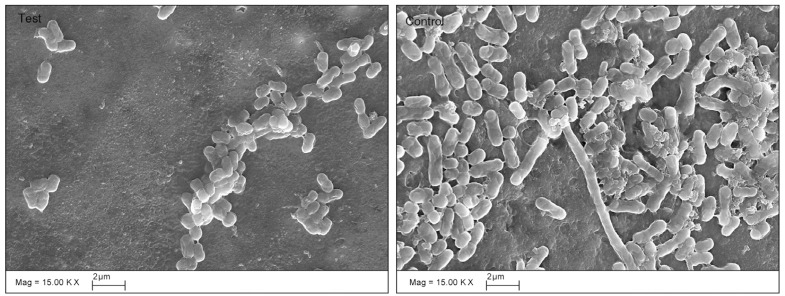
SEM images of biofilm (×15,000). Small clusters of bacterial cells were observed as isolated groups on the dentine surface treated with chlorhexidine, whereas a mono-layer of sparse biofilm was observed in the control group.

**Figure 3:**
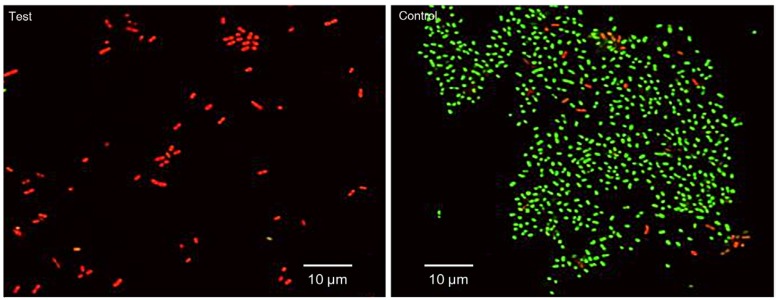
CLSM images of biofilm (×3,000). The green-fluorescent SYTO® 9 stain penetrated healthy bacterial cells and labelled both live and dead bacteria. The red-fluorescent propidium iodide stain penetrated only bacteria with damaged membranes, reducing the SYTO® 9 fluorescence. Dead bacteria with damaged membranes that fluoresced red dominated the test group, whereas live bacteria with intact membranes that fluoresced green dominated the control group.

 Table 1 shows the bacterial count, in log CFU, for both the test and the control group after the seven days of the experiment. Streptococci, Lactobacilli, Actinomycetes and the total bacterial counts were significantly lower in the test group than in the control group. The SEM images of the roots after seven days of incubation (Fig. 2) also showed differences between the test and control groups. In the test group, no biofilm was found, only small clusters of bacterial cells were observed as isolated groups, whereas a mono-layer of sparse biofilm was observed in the control group. In the CLSM images (Fig. 3), red (dead) cells clearly dominated the green (live) cells in the chlorhexidine treatment groups, indicating that the effects of chlorhexidine on bacteria viability was significant. The red to green (dead to live) ratios of the bacteria in the test group was 26.21±13.3 to 0.02±0.01, showing a significant increase of red cells with chlorhexidine applications (p=0.02).

**Table 1:**
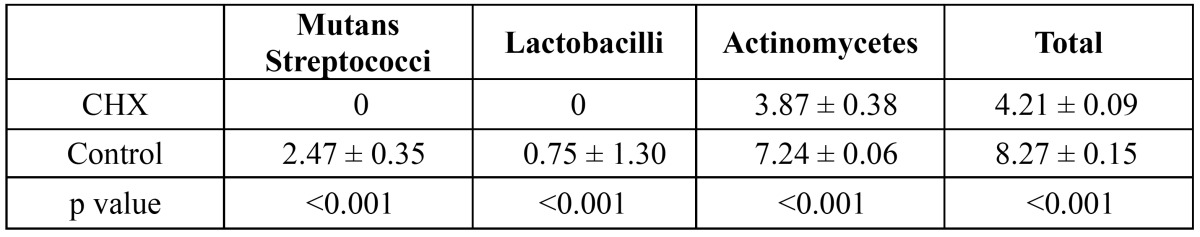
Log CFU from in-test group and control group (N=6).

-Hard tissue characteristics 

**Figure 4:**
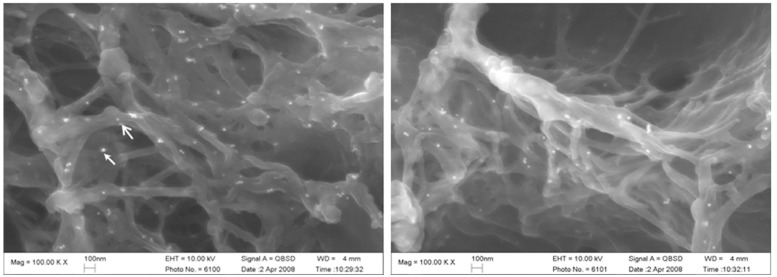
SEM image of Type I collagen and proteoglycans with immunocytochemical staining (×100,000). Type I collagen (30 nm particles, labeled by stealth arrow) and chondroitin 4/6 sulfate (15 nm, labeled by open arrow) with colloidal gold seen as bright spots.

As  Table 2 shows, the log [amide I: HPO42-] of the human root surface under the oral biofilm treated with chlorhexidine was significantly lower than that of the control group (p=0.022). (Fig. 4) shows an SEM image of the immunocytochemical staining of the type I collagen (30 nm particles) and proteoglycans (15 nm particles); the colloidal gold is seen as bright spots. In the control group, there were structural modifications to the collagen network (such as collapse, swelling, and branching) of the root surfaces that had not received the chlorhexidine treatment. The test group, treated with chlorhexidine, showed little damage to the collagen. ( Table 2) and (Fig. 4) show that there were significant differences in the number of 30 nm gold particles representing type I collagen detected in the test and control groups (p<0.05). The distribution of proteoglycans, as represented by the number of 15 nm gold particles, was higher in the test group than in the control group (p<0.05).

**Table 2:**
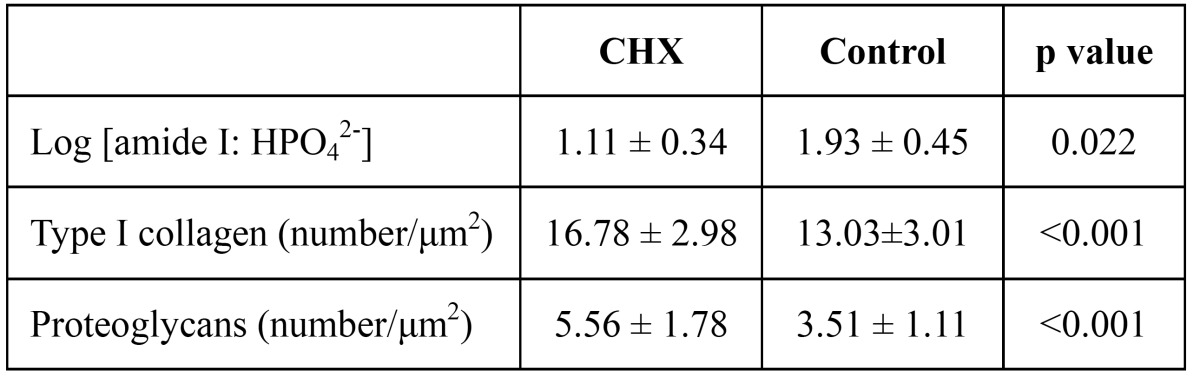
Log FTIR and collagen/proteoglycans intensity ratio (N=6).

## Discussion

When considering the outcome of this study, the limitations of such an in vitro study must be taken into account. The artificial mouth used in this study is one of the most sophisticated model systems used for caries research; it mimics the in vivo environ-ment in terms of temperature, humidity, sucrose supply and salivary rate. However, it has its own stable environment that is different from the in vivo environment. In addition, there are variations in the composition of biofilms between individuals. The variations apply not only to species composition, but also to baseline metabolic activities and to the consortia response to various growth substrates or antimicrobials. A study of the temporal changes in bacterial population using denaturing gradient gel electrophoresis found bacterial community diversity in the saliva of different individuals (20). The present study collected bacterial samples from three individuals. The results cannot be interpreted as representative of the bacteria existing in a large population.

The consortia biofilm of four species of oral bacteria on bovine enamel and dentine (21) and human dentine (22) successfully reproduced caries in an artificial mouth. These studies showed that a microcosm can be generated, creating a laboratory subset that evolved from a natural system (23). After five days, the microcosm formed using mixed salivary bacteria is similar to oral biofilms and also varies between individuals (24). A stabilized microcosm is thus one of the better simulations of in vivo oral biofilm. Therefore, the current study used mixed salivary bacteria from individuals to form a microcosm.

The antibacterial activity of chlorhexidine has been studied using various models. It has been shown that the minimum concentration of chlorhexidine required to kill bacteria in a biofilm is considerably higher (10-100 times) than the amount required to kill the bacteria in a suspension. Theoretically, the inhibition of caries by an antimicrobial agent can be achieved by the inhibition of acid production or the formation of dental plaque. In the present study, chlorhexidine was supplied from the beginning of the experiment. It was found that chlorhexidine had a higher suppression on the activities of selected cariogenic bacteria, including streptococci and Lactobacilli, than on the activities of Actinomyces. This study also found that in the presence of chlorhexidine, the bacteria failed to form biofilm when compared with the control group, in which a mono-layer of biofilm was formed. Similar results have been reported in previous in vitro studies, which found that pulses of chlorhexidine led to a selective long term suppression of Streptococci mutans (25,26). Moreover, it was also found that chlorhexidine was more efficacious against streptococci and Lactobacilli than against Actinomyces. In a previous clinical trial, an increase in the number of Actinomyces viscosus/naeslundii was noted after treatment with chlorhexidine varnish (27).

This study also found a significantly higher dead/live ratio value with chlorhexidine application, suggesting that chlorhexidine could exert an antimicrobial effect on biofilm. Computer software was used to differentiate colors (red/green) and areas to make quantitative analysis of confocal images possible (28). The results, however, may vary due to the uneven distribution of bacteria in different thicknesses of biofilm. Furthermore, the quality of the images could be affected by conditions such as brightness, white balance and contrast. Therefore, this method was only used to support the analysis, but conclusions cannot be draw solely based on these results.

Once the apatite crystallite of tooth tissue is dissolved, collagen fibers in the tooth are exposed and amide I is released as a by-product of collagen breakdown. The mineral density of the root surface can be assessed with the HPO42- band in the FTIR spectrum (29). The log [amide I: HPO42-] value has been used in previous laboratory studies as an indicator of the extent of demineralization of tooth tissue; the larger log values correspond to a greater extent of demineralization (22,30). The FTIR results of this study showed there was less demineralization of root surface in the chlorhexidine treatment groups than in groups without treatment. The use of a double-labeling technique permitted an investigation of both the presence and the distribution of type I collagen fibrils and proteoglycans on the root surface (31). These are the main structural components of the root surface network (32). The number of gold particles representing type I collagen and proteoglycans were higher in the test group than in the control group. With the chlorhexidine application, the bacteria failed to form a biofilm and the harmful effects of the bacteria on root surfaces were thus reduced in the test group and the collagens in the root surface were protected.

In conclusion, Chlorhexidine suppressed the activities of selected cariogenic bacteria in biofilm generated from human saliva; it also protected the mineral and organic content of human tooth roots from caries attacks. It can be a useful agent to prevent root caries in particular those patients with high caries risk. Further clinical trials should be performed to substantiate its effectiveness in root caries prevention.
